# Electroacupuncture stimulation improves cognitive ability and regulates metabolic disorders in Alzheimer’s disease model mice: new insights from brown adipose tissue thermogenesis

**DOI:** 10.3389/fendo.2023.1330565

**Published:** 2024-01-12

**Authors:** Ting Li, Junjian Tian, Meng Wu, Yuanshuo Tian, Zhigang Li

**Affiliations:** ^1^School of Acupuncture-Moxibustion and Tuina, Beijing University of Chinese Medicine, Beijing, China; ^2^Dongzhimen Hospital, Beijing University of Chinese Medicine, Beijing, China

**Keywords:** Alzheimer’s disease, metabolic, brown adipose tissue, thermogenesis, tau pathology, electroacupuncture

## Abstract

**Background:**

Metabolic defects play a crucial role in Alzheimer’s disease (AD) development. Brown adipose tissue (BAT) has been identified as a novel potential therapeutic target for AD due to its unique role in energy metabolism. Electroacupuncture (EA) shows promise in improving cognitive ability and brain glucose metabolism in AD, but its effects on peripheral and central metabolism are unclear.

**Methods:**

In this study, SAMP8 mice (AD model) received EA stimulation at specific acupoints. Cognitive abilities were evaluated using the Morris water maze test, while neuronal morphology and tau pathology were assessed through Nissl staining and immunofluorescence staining, respectively. Metabolic variations and BAT thermogenesis were measured using ELISA, HE staining, Western blotting, and infrared thermal imaging.

**Results:**

Compared to SAMR1 mice, SAMP8 mice showed impaired cognitive ability, neuronal damage, disrupted thermoregulation, and metabolic disorders with low BAT activity. Both the EA and DD groups improved cognitive ability and decreased tau phosphorylation (p<0.01 or p<0.05). However, only the EA group had a significant effect on metabolic disorders and BAT thermogenesis (p<0.01 or p<0.05), while the DD group did not.

**Conclusion:**

These findings indicate that EA not only improves the cognitive ability of SAMP8 mice, but also effectively regulates peripheral and central metabolic disorders, with this effect being significantly related to the activation of BAT thermogenesis.

## 1 Introduction

It is well known that Alzheimer ‘s disease (AD) is a neurodegenerative disease. The aggregation of extracellular amyloid plaques and formation of neurofibrillary tangles by hyperphosphorylated tau protein in neurons are the main neuropathological features closely related to central metabolic defects (1). Recent research has shown that the basic determinants of metabolism, such as a decline in brain glucose metabolism (2) and central insulin resistance, are the characteristics of AD intrinsic metabolic disorder (3), which is inextricably related to glucose metabolism and insulin sensitivity in the peripheral circulation (4, 5). Based on the widespread application of positron emission tomography (PET) scanning in medical research (6), several studies have confirmed the pathological characteristics of brain glucose metabolism disorders in patients with AD (7). Effective utilization of glucose in the brain is crucial for neuronal function and central activity. Impaired glucose utilization may be a key factor in the pathogenesis and is directly related to cognitive impairment in patients with AD.

Epidemiological studies have shown that AD is highly prevalent in the older adult population after 65 years of age, and its prevalence increases exponentially with age (8, 9). It is conceivable that the incidence of AD will continue to rise (10, 11), with the progress of social aging and the increase of human life expectancy. Theories of aging may explain the origin of AD under systematic considerations. Age also plays an important role in energy metabolism. With an increase in age, the metabolic rate of the body slows down (12), the risk of an imbalance between heat generation and consumption increases (13), and the ability to maintain the core temperature of the body decreases because of the dissipation of more heat due to the reduction of peripheral vascular contraction (14, 15). A previous study showed that, compared to young people, the body temperature of older adults is relatively different, and the amplitude of the circadian rhythm is slightly decreased (16–18). A systematic review found that the normal body temperature of adults over 60 years old was lower than that of the young (19), and the comprehensive temperature data of the rectum, mouth, armpit and ear showed that the average body temperature of older adults was 0.3°C lower than the acceptable normal hypothermia value (20). Studies have suggested that the loss of thermoregulation observed in elderly individuals may be caused to some extent by low BAT activity (21). Although this has not been confirmed in older volunteers, the role of BAT in accelerating thermogenesis cannot be ignored. It is generally believed that aging and thermal regulation defects are common phenomena in older adults and patients with AD (22).

As a uniquely thermogenic tissue in mammals (23–26), BAT decouples mitochondrial ATP synthesis from fuel oxidation via an uncoupling protein (UCP1), releasing and spreading all the energy generated by glucose and triglyceride metabolism in the matrix into the whole body in the form of heat. Thus, it can regulate body temperature, improve glucose (27–29) and lipid metabolism (30), and regulate insulin resistance (31, 32) and has been considered a valuable potential target for the treatment of peripheral and central metabolic disorders (22, 32–36), such as Alzheimer’s disease, in which age-associated thermoregulatory deficits contribute to energy metabolic failure.

Electroacupuncture (EA) is a modern method of acupuncture based on the meridian theory of traditional Chinese medicine (TCM) and is widely used in clinical practice. EA involves microbiological stimulation through the administration of small electric currents at selected acupoints along the meridians to prevent and treat diseases. GV20 and GV29 are specific governor-vessel acupoints. According to the TCM meridian theory, “governor meridian is the sea of yang vessels,” and it regulates the yang qi (vital energy) of the whole body. The role of yang qi is to warm the whole body, a function that is very similar to the BAT thermogenic function of maintaining core body temperature.

Our previous research has proven that electroacupuncture significantly enhanced glucose metabolism in the hippocampus (37, 38) and improved cognitive function by delaying pathological deposition through AKT/GSK3β signaling (39). However, the mechanism by which EA regulates brain glucose metabolism has not yet been elucidated, and it is unclear whether it affects both peripheral and central metabolic reactions. Therefore, considering the unique role of BAT, we visualized and qualitatively analyzed the thermogenic activity of BAT, observed the glucose and triglyceride content in the peripheral serum of SAMP8 mice, analyzed the HOMA-IR index, quantitatively analyzed the key proteins of the central insulin pathway, and performed a correlation analysis to elucidate the effect of EA on BAT thermogenesis and peripheral-central metabolism. The current study reveals the effects of EA on AD from different perspectives and provides a basis for further elucidation of the pathological mechanisms and treatment of AD.

## 2 Materials and methods

### 2.1 Experimental animals

Eight-month-old male SAMP8 (accelerated aging mice/prone 8) and homologous anti-aging SAMR1 mice (accelerated aging mice/resistance 1) were purchased from Beijing Huafukang Biotechnology Co. Ltd. (animal lot: SCXK [Beijing] 2019-0008). The mice were raised in a single cage in a barrier environment animal room at the Beijing University of Chinese Medicine. The animals were given adaptive feeding for 5 days before the experiment, temperature at 22 (± 2) °C, humidity at 45 (± 5) %, a 12h/12h day and night cycle, and free access to food and water. All experimental procedures and animal welfare were approved by the Animal Ethics Committee of Beijing University of Chinese Medicine (Ethics number: BUCM-2021110810-4174).

### 2.2 Animal grouping and intervention

Forty-five SAMP8 mice were randomly divided into three groups: the AD model group (AD group), Donepezil Drug group (DD group), Electro-Acupuncture Group (EA group). Fifteen SAMR1 mice served as the normal control group (NC group).

The NC and AD groups were used as experimental controls under the same feeding conditions without any treatment. However, they were fixed with a self-made mouse sleeve at the same time each day for 28 days.

The mice in the DD group were gavaged with donepezil at a dose of 0.65 mg/kg, crushed when used, and dissolved in 0.9% saline (40).To ensure the same processing conditions, the mice were fixed with a self-made mouse sleeve at the same time each day for 28 days.

The mice in the EA group were fixed inactively with a self-made mouse sleeve that did not affect breathing. Two disposable sterile acupuncture needles (size: 0.25 mm × 13 mm, ZY500, Beijing Zhongyan Taihe Medical Instrument Co. Ltd., China) were obliquely inserted 0.5 cm deep into the GV20 and GV29 acupoints. GV20 was located at the midpoint between the auricular apices, whereas GV29 was located midway between the medial ends of the two eyebrows. The needle handle was connected to an EA instrument (SDZ-V; Suzhou Medical Supplies Factory Co., Ltd. China). The frequency of the instrument was adjusted to 2 Hz and the current intensity was 1 mA. The needle handle vibrated slightly, whereas the mice were quiet and did not struggle. GV26 used the prick method and did not retain needles. EA was administered for 20 min at the same time every day for 28 days.

### 2.3 Core body temperature monitoring and acquisition of infrared thermal imaging

Core body temperature was monitored before and after the intervention using an Intelligent Digital Rectal Thermometer (TH212, China Communications Construction Technology Development Co., Ltd., China). The thermometer was inserted gently approximately an inch into the rectum, taking care not to insert it too far. It was kept in place for about a minute or until it beeped, indicating the reading was complete. The temperature displayed on the thermometer was recorded, and the thermometer was then cleaned according to the manufacturer’s instructions. These steps were repeated two more times to measure the rectal temperature, ensuring that the thermometer was properly cleaned before each subsequent measurement. Each measurement was conducted within the same time range (2:00-4:00 pm) to ensure consistency and accuracy. Finally, the average value obtained from the three recorded temperatures was used as the core temperature value for each mouse.

After the 28-day intervention period, all mice were allowed to move freely above the cage lid. Meanwhile, an infrared camera lens was placed 0.5 m above the mice to shoot thermal images of their backs (hair on the backs was removed one day before shooting).

Infrared thermal imaging is a technique used to determine surface temperature according to the physical law of radiation transfer. Medical infrared thermal imagers focus on the invisible infrared radiation energy emitted by an object and subject it to photoelectric conversion to generate processed electrical signals. The electrical signal is then simulated, amplified, and converted into a digital image signal. An image showing the thermal characteristics of an object was presented on the corresponding display device. A warm (bright) color represents a high-temperature region, whereas a cool (dark) color represents a low-temperature region. The intensity of the infrared radiation emitted by an object is primarily a function of its temperature. Infrared thermal imaging has been used to study surface temperatures in various mammals (41–43), and some researchers have used it in TCM research on acupuncture meridians and acupoints (44–47).

A Testo 865 handheld infrared thermal imager (Germany) was used to display the distribution of the skin surface temperature fields. The imager is a 160×120-pixel infrared pixel detector that can detect objects sized< 0.3 mm. The imager can detect temperatures of −20°C to 280°C. The imager is continuously adjustable, with thermal sensitivity of<0.12°C. The SUPPER infrared superpixel (320×240 pixels) has an image enhancement function that captures images through precise displacement, a built-in visible light shooting component with LED lighting, and an autofocus capacity (minimum focus, 0.5 m). It shows the isothermal and low, high, and average values of the regions. Moreover, the reflectivity and reflection of the detected temperature can be set manually. Infrared thermal images can be accurately analyzed on a computer using the IRSOFT professional infrared analysis software. The emissivity of mouse skin (e = 0.97), room temperature (23–25°C), relative humidity (50–60%), and detection distance (0.5 m) were the same for all groups.

### 2.4 Morris water maze

The Morris water maze (MWM) trial was conducted for 6 days and the hidden platform trials was conducted for the first 5 days. The platform was placed in the center of the southwest (SW) quadrant, slightly below the water surface. The mouse finds a platform to escape. If a hidden platform was successfully identified, the video acquisition system stopped automatically. If they were not found within 60 s, the mice were guided to find the platform and allowed to stay on it for 10 s. The escape latency and swimming speed were recorded. The hidden circular platform in the SW quadrant was removed for the probe trial. The mice were placed in water gently facing the wall of the pool, and the swimming path of the mice was observed within 60 s. The platform crossover number and percentage of time spent in the target quadrant were analyzed.

### 2.5 Nissl staining

The mice were anesthetized by an intraperitoneal injection of pentobarbital (80 mg/kg body weight) for the sample collection. The mouse brain tissue was completely removed, fixed with paraformaldehyde, embedded in paraffin, then sliced, dewaxed, and stained with 0.5% toluidine blue aqueous solution (RY-0004, Beijing Zhongke Wanbang Biotechnology Co. Ltd., China). Stained tissues were made transparent, mounted, and observed under a microscope (KF-PRO-120, China).

### 2.6 Hematoxylin-eosin staining

Brown adipose tissue was embedded in paraffin, sliced, dewaxed, and stained with hematoxylin-eosin (RY-0002, Beijing Zhongke Wanbang Biotechnology Co. Ltd., China). The stained tissues were dehydrated, mounted, and observed under a microscope.

### 2.7 Immunofluorescence staining

Mouse brain tissues (3-mm thick) were collected, fixed with paraformaldehyde, and dehydrated using an ethanol gradient. The tissues were paraffin embedded and sliced into 4-micron thick slices with a slicing machine (Leica, Germany, model: RM2235). Immunofluorescence staining was performed. The primary antibody used was rabbit polyclonal P-tau (1:200; antibody number: bs-2368R, China). The secondary antibody used was Goat Anti-Rabbit IgG H&L Alexa Fluor 594 (1:500; antibody number: ab150080, China). Finally, the sections were stained with DAPI (YG-0001; Beijing, China). Fluorescence scanning was performed using ImageJ.

### 2.8 Enzyme-linked immunosorbent assays

Blood samples were collected by eyeball extraction. The centrifuge tube containing blood was centrifuged at 4°C and 10000 rpm for 5 min, and the supernatant was separated into serum. The serum was placed in a cryopreservation tube and stored in a refrigerator at -20°C. Serum glucose, triglyceride, and insulin levels were detected using a mouse enzyme-linked immunosorbent assay kit, according to the manufacturer’s instructions, and the absorbance was read.

### 2.9 Western blotting

Mouse tissues were removed from liquid nitrogen for rapid grinding in a precooled mortar. The ground powder and cell lysates were placed in Eppendorf tubes. After centrifugation at 4°C, tissue proteins were extracted, and protein quantification was performed using the bicinchoninic acid method. A 15% SDS-PAGE separation gel solution was prepared and 5% concentrated gel was added. The treated samples were placed in the sample holes of a concentrated gel in a predetermined order for electrophoresis. After the protein samples were electrotransferred, a polyvinylidene fluoride (PVDF) membrane (Millipore, Germany; product number: IPVH00010, batch number: R9PA20712) was taken out and sealed with 5% TBS-T skimmed milk powder at room temperature for 60 min. Next, the primary antibodies were added and blocked at 4°C overnight. After washing, the secondary antibodies were added and shaken at 37°C for 60 min. Finally, an enhanced chemiluminescence method was used to expose and develop the imprinting. Glyceraldehyde-3-phosphate dehydrogenase (GAPDH) was used as an internal control. The density of all WB bands was compared with that of the GAPDH band.

### 2.10 Statistics analysis

Statistical analyses were performed using SPSS 26.0 (SPSS/IBM 26.0; SPSS Inc., USA). Data are expressed as the mean ± SD ( $\bar{X}\pm S$ ). The hidden platform trial and swimming speed results were analyzed using a repeated-measures analysis. One-way ANOVA followed by the least significant difference (LSD) multiple-range test was used to compare normally distributed data and homogeneity of variance. The level of significance was set at *P<* 0.05. Pearson correlation analysis was used to analyze the correlation between variables.

## 3 Results

### 3.1 EA stimulation can improve the spatial learning and memory ability of AD model mice

**Figure 1:**
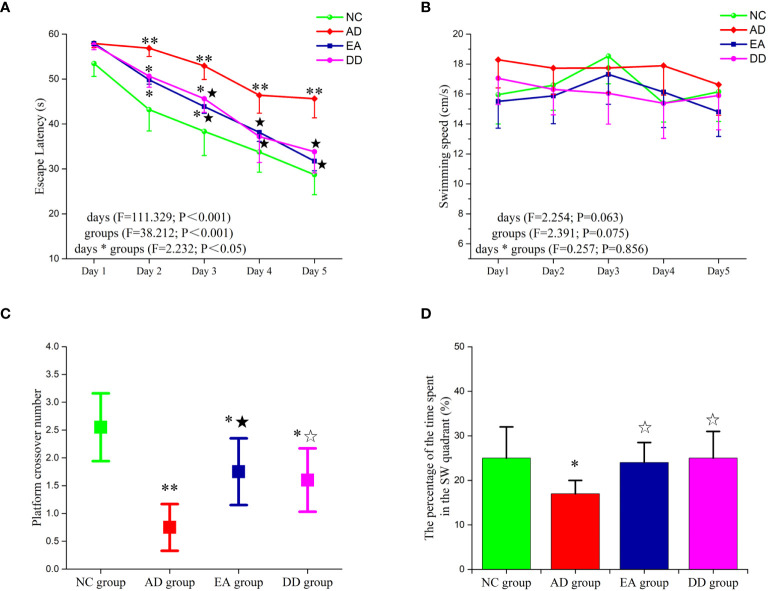
Results of MWM trial (n=10, mean ± SD) for each group. **(A, B)** Comparison of the escape latency and swimming speed of each group in the hidden platform trials. **(C, D)** Comparison of the platform crossover number and the percentage of time spent in the target quadrant of each group in the probe trial. Compared to the NC group, *P<0.05,**P<0.01 compared to the AD group, ^☆^*P*<0.05, ^★^*P*<0.01.

The results of the MWM test are shown in **Figure 1**. The escape latency of the NC, EA, and DD group decreased gradually from the second to the fifth day and decreased significantly from the third to the fifth day (*P<* 0.05 or *P<* 0.01). However, no significant change was observed in the AD group from the second day to the third day. Until the fourth day, escape latency in the AD group showed a downward trend. Compared to the AD group, the escape latency of the NC group was significantly lower (*P<* 0.05, *P<* 0.01). The escape latencies of the EA group and DD group were significantly lower than those of the AD group from the second day to the fifth day (*P<* 0.01). No significant differences were observed between the EA and DD groups. No significant differences in swimming speed were observed between groups.

The platform crossover number (**Figure 1C**) and the percentage of time spent in the target quadrant (**Figure 1D**) were significantly higher in the NC group than in the AD group (*P<* 0.05, *P<* 0.01, respectively). Compared to the AD group, the platform crossover number and percentage of time spent in the target quadrant in the EA and DD groups were higher by varying degrees (*P<* 0.05, *P<* 0.01). No significant differences in these measurements were observed between the DD and EA groups. This indicated that EA stimulation could improve the learning and memory abilities of SAMP8 mice, and its efficacy was comparable to that of the DD group.

Donepezil (DNP) is a well-established and effective treatment for Alzheimer’s disease (48). As an acetylcholinesterase inhibitor, it plays a crucial role in maintaining the activity of neurons involved in learning and cognition by inhibiting the breakdown of acetylcholine at the synapses (49). Additionally, DNP’s selective inhibition of central rather than peripheral ChEs is expected to reduce the incidence of adverse events, including peripheral metabolism issues (50). Given its good tolerability and safety profile, DNP was used as a positive control group in our study to examine the effective improvement of EA on the behavior of SAMP8 mice.

### 3.2 EA stimulation can improve neuronal morphology and reduce P-Tau levels of AD model mice

**Figure 2:**
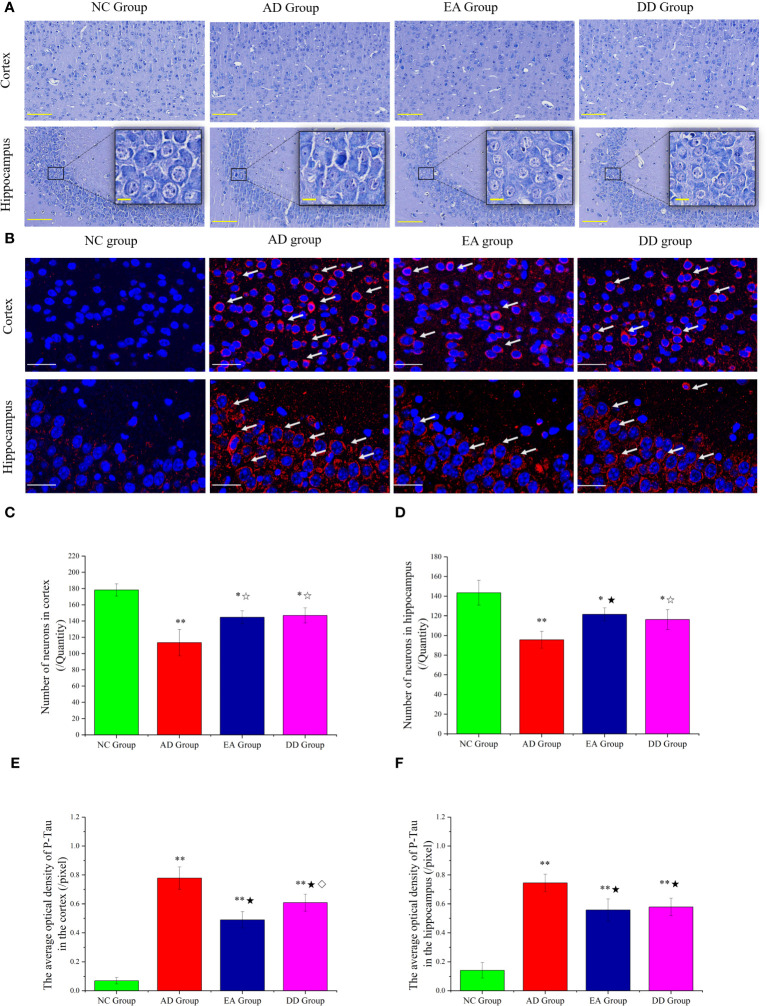
**(A)** Results of Nissl staining in the cortex and hippocampus of mice in each group (n=4) (The scale bar is 100 μm). The scale bar in the detailed enlarged picture is 25 μm. **(B)** Representative images of IF staining of P-tau (Thr231). P-tau (Thr231) are labeled with red fluorescence. The scale bar is 25 μm. **(C, D)** Comparison of the number of neurons in the cortex and hippocampus of each group (n=4). **(E, F)** Comparison of the mean optical density of P-tau in the cortex and hippocampus of each group (n=4). Supplement: Compared to the NC group, *P<0.05, **P<0.01 compared to the AD group, ☆P<0.05, ★P<0.01; compared to the EA group, ⋄P<0.05.

The results of the Nissl staining are shown in **Figure 2A**. In the NC group, the neurons were arranged regularly and densely and a clear and complete cell contour was observed. The neurons were rich in Nissl bodies, and the nucleus was full and satiated. In the AD group, nerve cells were severely damaged and a large number of cells were loosely arranged and disordered. Scattered cell debris, deep nuclear staining, and nuclear disappearance were also observed. The arrangement of nerve cells in the EA and DD groups was more regular than that in the AD group, and the cell contour was clear; however, the cell body and nucleus were still not as complete as those in the NC group were, and there were irregular cell bodies and nuclear staining.

Furthermore, undamaged and morphologically intact neurons are identified and counted, enabling quantitative analysis of their characteristics. The results are displayed in **Figures 2C, D**. Compared to SAMR1 mice, the number of neurons in SAMP8 mice (AD, EA, DD groups) was significantly reduced, with consistent trends observed in the hippocampus and cortex (P<0.01 or P<0.05). In comparison to the AD group, the number of neurons in the EA and DD groups showed a relative increase, suggesting that EA and DD interventions can effectively reduce neuronal damage.

We investigated the effect of EA stimulation on pathological tau protein by conducting immunofluorescence staining. **Figure 2B** demonstrates the merged staining results, where P-tau (Thr231) was labeled with red fluorescence and DAPI was labeled with blue fluorescence. The intensity of the staining corresponds to the level of protein expression. This staining technique allowed us to visualize and analyze the distribution of P-tau as well as assess any changes induced by EA stimulation.

In the cortex of the NC group, there was no significant expression of P-Tau, as indicated by the absence of red fluorescence. Conversely, in the AD group, there was a notable presence of red fluorescence, primarily concentrated in and around the neuronal cell body. When comparing the EA group and DD group to the AD group, the red fluorescence expression appeared relatively weakened in both treatment groups. We specifically analyzed the CA3 area of the hippocampus. The NC group showed no significant red fluorescence expression, indicating the absence of P-tau. In contrast, the AD group displayed a prominent increase in red fluorescence, indicating a high level of P-tau expression. Interestingly, both the EA and DD groups exhibited relatively weakened red fluorescence expression, suggesting that EA stimulation and Donepezil drug treatment may potentially contribute to a reduction in P-tau levels.

Upon further analysis (**Figures 2E, F**), we found that the average optical density in the cortex and hippocampus was significantly higher in the AD, EA, and DD groups compared to the NC group (P< 0.01). Furthermore, when comparing the AD group with the EA and DD groups, we observed a significant decrease in both the content and average optical density of P-tau in these groups(P< 0.01). Remarkably, the EA group displayed a more pronounced effect in reducing cortical P-tau levels compared to the DD group (P< 0.05). In summary, our findings indicate that EA stimulation has a substantial impact on reducing P-tau levels, particularly in the cortex.

### 3.3 AD model mice have peripheral metabolic disorders, which can be regulated by EA stimulation

**Figure 3:**
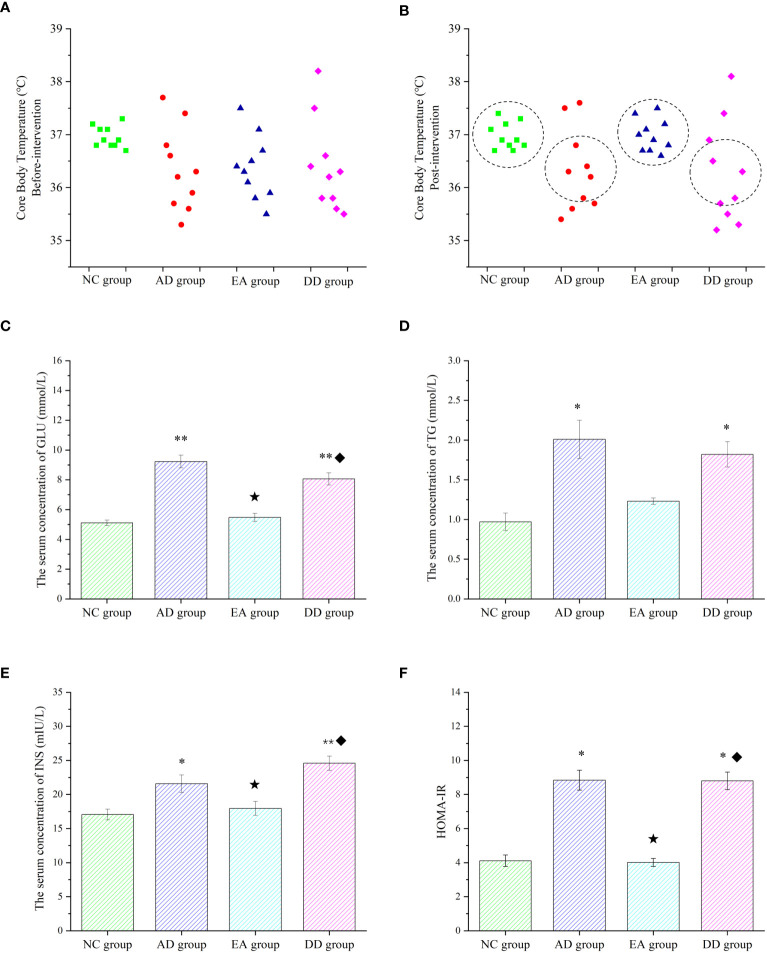
Peripheral metabolic results of each group. **(A, B)** Comparison of core body temperature before and after intervention in each group (n=10). **(C–F)** ELISA results of glucose, lipid, insulin, and HOMA-IR in peripheral blood (n=6). Compared to the NC group, *P<0.05,**P<0.01 compared to the AD group, ^★^*P*<0.01; compared to the EA group, ^♦^*P*<0.01.

Core body temperature is the most intuitive indicator of metabolic reactions. There was no significant difference in the core body temperature of each group before the intervention. However, the distribution range of the core temperature values of each group was compared. As shown in **Figure 3A**, the core body temperature of the NC group was concentrated at about 37°C, while the distribution of the AD, EA, and DD groups was irregular and discrete. The temperature was as high as 38°C and as low as 35.3°C, but most were distributed between 36-36.5°C. The overall core temperature distribution was lower than that in the NC group. After the intervention, as shown in **Figure 3B**, there was still no significant difference in the core temperature between the groups (**Supplementary Material**). However, the body temperature distribution of the AD and DD groups was still discrete, whereas the distribution of the EA group showed a concentrated trend, close to the distribution trend of the NC group. **Figure 3C** shows the serum glucose (GLU) levels in each group. The GLU level of the NC group was within the ideal range, whereas the AD group was higher than that of the NC group (*P<* 0.01). Compared to the AD group, the serum GLU level in the EA group was significantly lower (*P<* 0.01), but there was no significant change in the DD group. Serum triglycerides (TG) in each group are shown in **Figure 3D**. Serum TG levels in the AD group were significantly higher than those in the NC group. Compared to the AD group, the serum TG content in the EA group was slightly reduced, but the difference was not statistically significant. Serum insulin (INS) levels are shown in **Figure 3E**. It was found that, compared with the NC group, the INS content of the AD group was higher, and the INS content of the EA group was not significantly different from that of the NC group. The INS in the EA group was significantly lower than that in the AD group (*P<* 0.01).

Insulin resistance (IR) was analyzed by evaluating the homeostasis model assessment (HOMA) index, and HOMA-IR levels were calculated using the following equation (51): (fasting blood insulin [mIU/L] × blood glucose [mmol/L])/22.5. The HOMA-IR values in each group are shown in **Figure 3F**. Compared with the NC group, the AD group was significantly higher (*P<* 0.05). The HOMA-IR values were significantly lower in the EA group than in the AD group (*P<* 0.01). However, the levels of serum GLU, TG, INS, and HOMA-IR in the DD group were higher than those in the NC group were, but not different from those in the AD group. This indicates that AD model mice have peripheral metabolic disorders, including thermoregulation disorders, which can be regulated by EA stimulation rather than by donepezil.

### 3.4 EA stimulation could promote BAT thermogenesis

**Figure 4:**
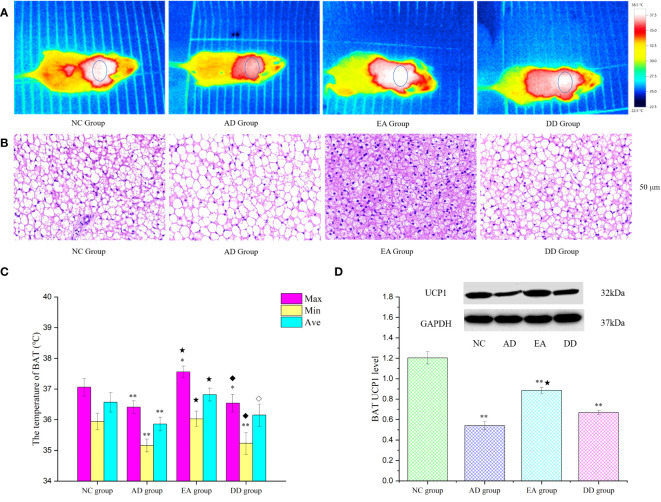
The results of BAT thermogenesis in each group. **(A)** The representative infrared thermal image of each group (n=10). **(B)** BAT HE results of each group (n=4) (50 μm). **(C)** Comparison of BAT skin temperature in each group (n=10). **(D)** Western Blot representative bands and statistical results of UCP1 protein in BAT (n=6). Compared to the NC group, *P<0.05,**P<0.01 compared to the AD group, ^★^*P*<0.01; compared to the EA group, ^⋄^*P*<0.05; ^♦^*P*<0.01.

Brown adipose tissue (BAT) is the only non-shivering thermogenic organ in mammals and plays an irreplaceable role in regulating glucose and lipid metabolism and maintaining energy homeostasis (52). To explore whether the improvement of thermoregulation and peripheral metabolism by EA stimulation is related to BAT, we analyzed the skin temperature of the BAT area by infrared thermography and the hematoxylin and eosin (HE) morphology of BAT tissue, and quantitatively studied the thermogenic protein UCP1. **Figure 4A** represents an infrared thermal image of each group. The small elliptical circle refers to the area in which the BAT is located. The warm (bright) and cold (dark) colors represent the high-temperature and low-temperature regions, respectively. The infrared thermal image shows that the small elliptical circle was red in the AD group, white in the NC group, and white and bright in the EA group, indicating that the skin temperature in the BAT area of the AD group was lower than that of the NC and EA groups. We statistically analyzed the maximum, minimum, and average skin temperatures in this small elliptical circular area. The results are shown in **Figure 4C**. Compared to the NC group, the highest and lowest temperatures in the BAT region of the AD and DD groups were significantly lower (*P<* 0.01), whereas the highest temperature in the EA group was higher. Compared with the AD group, the lowest, highest, and average temperatures in the EA group were significantly higher (*P<* 0.01), whereas there was no significant difference in the DD group.

The BAT HE staining results are shown in **Figure 4B**. H&E staining of the NC group revealed single, small lipid droplets in BAT and larger lipid droplets in the AD group. The BAT HE group showed a multilocular stimulation morphology. However, the DD group was similar to the AD group. **Figure 4D** displays representative bands and statistical results of UCP1 protein in BAT through Western Blot analysis. The UCP1 content in the AD, EA, and DD groups was found to be significantly lower than that in the NC group (P< 0.01). However, a notable increase in UCP1 content was observed in the EA group compared to the AD group. These findings support the notion that EA stimulation promotes BAT thermogenesis.

### 3.5 EA stimulation up-regulated the content of central insulin receptor protein (IRS-1), promoted the phosphorylation of AKT

**Figure 5:**
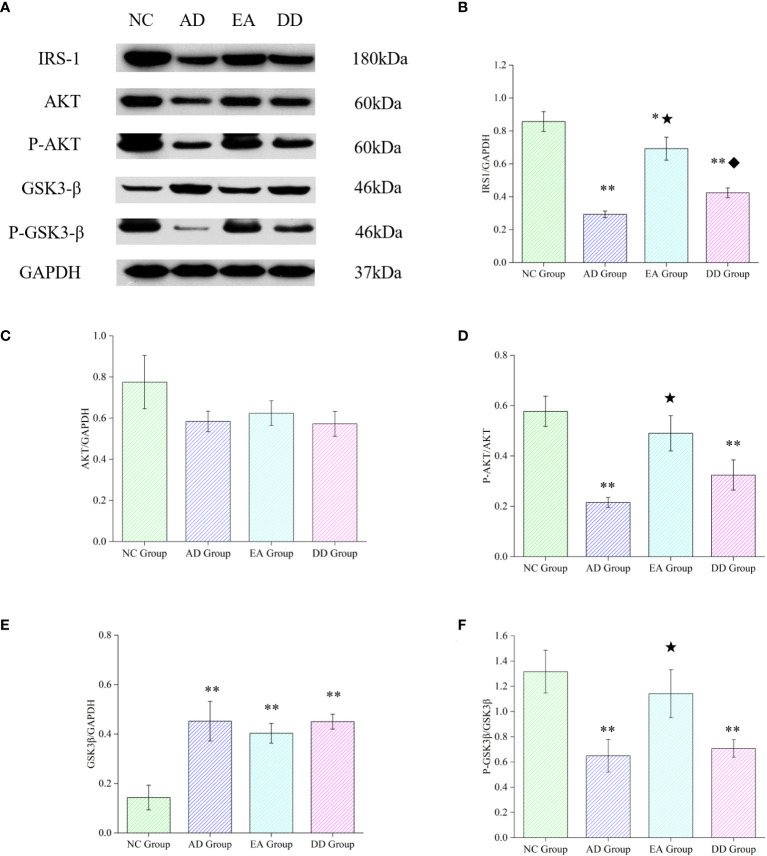
Results of key protein levels of the central insulin pathway and pathological tau protein in each group. **(A)** Western Blot representative bands of key proteins of the central insulin pathway. **(B-F)** Comparison of the relative expression of key proteins of the central insulin pathway in each group (n=6). Compared to the NC group, *P<0.05,**P<0.01 compared to the AD group, ^★^*P*<0.01; compared to the EA group, ^♦^*P*<0.01.

To further investigate whether the mechanism of EA stimulation in AD is related to the central insulin pathway, we analyzed the relative expression of key proteins in the cortical insulin pathway. As shown in **Figure 5**, compared with the NC group, the relative expression of IRS1, P-AKT, in the AD, EA, and DD groups were significantly lower than those in the NC group (*P<* 0.01 or *P<* 0.05), while the relative expression of GSK3β was higher than that in NC group (*P<* 0.01). There was no difference in the relative expression of AKT between groups. Compared to the AD group, EA stimulation significantly upregulated the relative expression of IRS1, P-AKT and P-GSK3β (*P<* 0.01). This indicates that EA stimulation upregulates the content of central IRS-1 and promotes AKT and GSK3β phosphorylation.

### 3.6 Effects of peripheral metabolism on key proteins of the central insulin pathway and their relationship with UCP1

**Figure 6:**
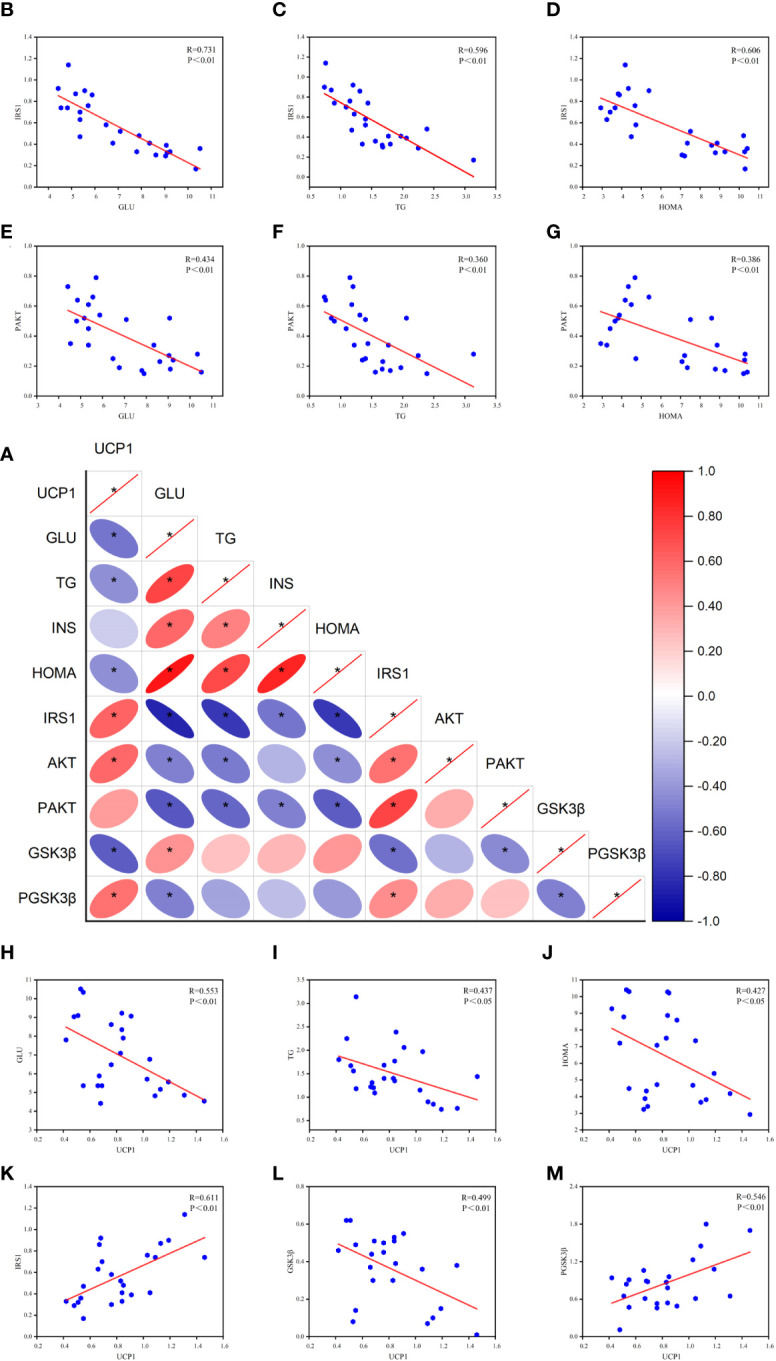
Results of Pearson correlation between peripheral metabolism and key proteins of the central insulin pathway. **(A)** Correlation heat map. **(B-D)** Pearson correlation analysis of GLU, TG, HOMA, and IRS-1. **(E-G)** Pearson correlation analysis of GLU, TG, HOMA, and P-AKT. **(H-J)** Pearson correlation analysis of GLU, TG, HOMA, and UCP1. **(K-M)** Pearson correlation analysis of UCP1 and IRS-1, GSK3β, P-GSK3β (R value and p-value given). *P<0.05.

Based on the above experimental results and the theory that peripheral and central metabolisms are closely related (4), we analyzed the Pearson correlation between peripheral glucose, lipid, HOME-IR, and key central insulin pathway proteins (**Figure 6**). We found a significant negative correlation between serum GLU and TG levels and central IRS-1 and P-AKT protein levels, especially with IRS-1 and HOMA-IR. This suggests that peripheral hyperglycemia, hyperlipidemia, and insulin resistance may be associated with decreased activity of central IRS-1 and P-AKT proteins. Based on these findings, we can infer that peripheral metabolism affects key proteins of the central insulin pathway, thereby influencing central insulin sensitivity.

Furthermore, the correlation analysis reveals a negative correlation between UCP1 and peripheral glucose, lipid, and HOMA-IR levels. This suggests that the activation of brown adipose tissue is associated with the utilization of glucose and lipids. Additionally, UCP1 shows a positive correlation with central IRS-1, indicating that the activation of brown adipose tissue has a positive effect on IRS-1 expression. Moreover, UCP1 exhibits a negative correlation with GSK3β and a positive correlation with P-GSK3β, suggesting a potential association between UCP1 and GSK3β activity. These correlation analysis results provide important insights into understanding the impact of peripheral metabolism on the central insulin pathway and lay the foundation for further research.

## 4 Discussion

The MWM test is an important means of evaluating the learning and spatial memory abilities of experimental animals. It is the main method used for behavioral testing in biological research because of its high reliability (53). In this study, the MWM test was used to evaluate the effect of EA on cognitive ability in an AD mouse model. The results of the hidden platform experiment revealed no significant differences in the swimming speed of each group, indicating that the swimming ability of each group was similar. The escape latency of the NC group was always lower than those of the AD, EA, and DD groups, indicating that the spatial learning ability of SAMR1 mice was higher than that of SAMP8 mice, which is consistent with the pathological changes in cognitive impairment in SAMP8 mice (54). The escape latency of the EA and DD groups was significantly lower than that of the AD group from the second to the fifth day, but the difference between the groups was not significant, indicating that donepezil and EA intervention could improve the spatial learning ability of SAMP8 mice; however, the difference in therapeutic effectiveness was not significant. The results of the probe trial showed that EA had an effect similar to that of donepezil in improving the spatial memory of SAMP8 mice. The cognitive deficits of AD are directly related to neuronal damage (55). Nissl staining showed a large amount of neuronal damage in the cortex and hippocampus of SAMP8 mice, whereas EA and donepezil reduced neuronal damage.

Several studies have highlighted a strong relationship between AD and metabolic disorders. A study conducted by researchers from the University of Oxford involved a 15-year follow-up on 176,000 participants without dementia. The study found that metabolic syndrome was associated with a 12% increased risk of developing dementia (56). These findings were corroborated by a research team from South Korea who also observed a connection between metabolic disturbances and an elevated risk of dementia (57). Recent studies have shown that a decline in brain glucose metabolism and central insulin resistance are considered characteristic features of metabolic disruption in AD patients (3) Disrupted brain glucose metabolism is a persistent feature throughout the pathological process of AD (5), and it may be a key factor in the disease’s mechanisms, triggering a cascade of reactions leading to neuronal degeneration and cognitive impairment in AD patients. Furthermore, this disruption is closely associated with peripheral glucose metabolism and insulin sensitivity (4).

Aging is widely considered as one of the principal risk factors to the pathological changes in AD (22, 58). Age plays a significant role in energy metabolism, as metabolic rates tend to slow down as individuals grow older (12).This imbalance in energy production and expenditure disrupts the equilibrium, leading to a decline in the ability to maintain core body temperature (14).The defect of the body temperature regulation mechanism in older adults is accompanied by a sharp increase in the incidence of AD (19, 59). This suggests the possibility that age-related thermoregulatory defects lead to energy failure during AD pathogenesis (22, 58). Therefore, AD is a complex age-related neurodegenerative disease, associated with central and peripheral metabolic anomalies, such as impaired glucose utilization and insulin resistance (22). Therefore, aging is the principal risk factor and the most significant physiological feature of AD. It is accompanied by a decline in metabolism. On the other hand, metabolic diseases, such as diabetes, are characterized by glucose and insulin dysfunctions, and have been documented to be associated with an increased risk of developing AD.

To investigate the role of EA in AD energy metabolism, we conducted an in-depth study. Our study examined the core body temperature of each group and found that the mean value was not significantly different between SAMR1 and SAMP8 mice before and after the intervention. However, analysis of the body temperature distribution in each group showed that the distribution of SAMP8 mice was discrete before the intervention. Following the intervention, the distribution trend in the EA group showed clear changes, and the overall trend was similar to that in the NC group. These results indicate that SAMP8 mice have thermoregulation disorders that mainly manifest as overall hypothermia, but individual differences are large (16). Body temperature is the oldest known human metabolic parameter. To date, no consensus has been reached on the association between the pathological diagnosis of AD and the mean body temperature. It has been reported that there is no difference in average body temperature between patients with AD and normal controls (60). Some studies have suggested that patients with AD have a lower core body temperature (61), whereas others have reported opposite results (62). These inconsistencies depend on how and when the temperature is measured, as well as the stage of the disease and the associated motor or neuropsychological symptoms. Patients with AD often experience agitation and behavioral disorders that may affect their body temperature. The discrete distribution of core body temperature in SAMP8 mice also illustrates an obstacle to its thermoregulatory mechanism, which is more closely related to AD than to simple temperature differences.

A study of GLU, TG, and INS in peripheral serum found that SAMP8 mice had obvious metabolic disorders, manifested as hyperglycemia, hyperlipidemia, and hyperinsulinemia. These results are also consistent with a previous study in which SAMP8 mice showed age-related insulin resistance (63). This suggests that the SAMP8 strain is the best rodent model for studying age-related metabolic complications. It is also associated with energy metabolism disorders. EA regulated these disorders, whereas DD showed no effect on peripheral metabolic disorders.

When talking about thermoregulation and peripheral glucose and lipid metabolism, the role of BAT must be considered. BAT is a key component of mammalian non-shivering thermogenesis that maintains core temperature and energy consumption (23–26). Previous studies in rodents have shown (22) that the regulation of the internal body temperature by exposing BAT to cold directly affects AD pathology. Although cold adaptation is not suitable for elderly individuals, this study provides the first evidence that chronic BAT stimulation may be a valuable strategy for treating AD. Subsequent studies have found that tau phosphorylation is extremely sensitive to temperature (64) and that BAT stimulation can antagonize tau phosphorylation (65, 66). In this study, we used infrared thermal imaging technology to evaluate the effect of EA on BAT thermogenic activation and quantitatively analyzed UCP1 thermogenic protein. Our results support the idea that EA stimulation promotes BAT thermogenesis, although no statistically significant increase in core body temperature was observed in mice. UCP1 is considered the main molecular driving force behind thermal regulation and energy homeostasis of BAT (67). Our results showed that although the content of UCP1 in the EA group was up-regulated, the content of UCP1 in the BAT of SAMP8 mice was still much lower than that in SAMR1 mice (*P<* 0.01), which also suggests that thermal regulation and metabolic disorders in SAMP8 mice may be related to low BAT activity.

Evidence suggests that reduced phosphorylation of insulin receptors and downstream substrates leads to IR in the brain. The PI3K/AKT-dependent pathway can improve cerebral insulin resistance and regulates tau phosphorylation in AD (68). Based on our immunofluorescence results for P-tau, we found that EA could inhibit the phosphorylation of tau in the cortex and hippocampus. Therefore, we quantitatively analyzed key proteins in the insulin pathway and found that EA significantly upregulated the content of IRS-1 and promoted AKT phosphorylation. This was consistent with the results of our previous acupuncture experiments based on APP/PS1 mice (39).

AD is a central peripheral metabolic disease with thermoregulation deficiency (22). Studies have shown that peripheral blood-based interventions can improve or even reverse related brain dysfunctions (69). In this study, we found a significant negative correlation between peripheral glucose, lipid, HOMA-IR, IRS-1, and p-AKT levels, indicating that peripheral metabolic disorders may influence the sensitivity of the central insulin pathway. EA can regulate peripheral metabolic disorders, significantly increase IRS-1 levels, promote AKT phosphorylation, and enhance insulin sensitivity. Further correlation analysis showed that the activation of BAT thermogenesis promotes peripheral glucose and lipid metabolism, regulates insulin resistance, and has a positive effect on IRS-1 upregulation. GSK3β is a downstream target of the PI3K/AKT signaling pathway and is involved in tau phosphorylation as a major tau kinase (70). The positive correlation between UCP1 and P-GSK3β suggests a potential connection between the activation of BAT thermogenesis and the activity of GSK3β. This relationship could potentially have a beneficial effect, helping to alleviate the negative effects caused by high levels of GSK-3β on the brain. Therefore, the mechanism by which EA reduces the abnormal phosphorylation of tau is likely to be attributed to the effect of BAT thermogenesis on overall metabolism. This suggests that intervention in peripheral metabolism may be an effective therapeutic strategy for AD.

## 5 Conclusion

In this study, we confirmed that EA improves cognitive function and neuronal morphology in SAMP8 mice. This is the first report that EA regulates thermoregulatory dysfunction and peripheral metabolic disorders in SAMP8 mice. We demonstrated for the first time that EA has an overall regulatory effect on peripheral-central metabolism, and proposed that this effect may be related to BAT thermogenesis.

Furthermore, AD is not a simple brain pathological disease but also a closely related peripheral and central metabolic disease. Therefore, a new perspective on the pathology of AD is required. From a holistic perspective, the periphery and center are viewed as a whole that can interact with each other for better understanding and treatment. EA is a method for understanding and treating diseases based on a holistic view of traditional Chinese medicine. This may explain why EA has a multitarget effect compared to donepezil.

## Data availability statement

The original contributions presented in the study are included in the article/**Supplementary Material**. Further inquiries can be directed to the corresponding author.

## Ethics statement

The animal study was approved by Animal Ethics Committee of Beijing University of Chinese Medicine (Ethics number: BUCM-2021110810-4174). The study was conducted in accordance with the local legislation and institutional requirements.

## Author contributions

TL: Conceptualization, Data curation, Formal analysis, Methodology, Writing – original draft, Writing – review & editing. JT: Data curation, Formal analysis, Methodology, Writing – original draft. MW: Data curation, Formal analysis, Methodology, Project administration, Writing – review & editing. YT: Project administration, Writing – review & editing. ZL: Conceptualization, Writing – review & editing.
