# Supplementary material for: Electroacupuncture stimulation improves cognitive ability and regulates metabolic disorders in Alzheimer’s disease model mice: new insights from brown adipose tissue thermogenesis
Source: Front Endocrinol (Lausanne). 2024 Jan 12;14:1330565. doi: 10.3389/fendo.2023.1330565 (PMC10811084; doi:10.3389/fendo.2023.1330565)
Supplement: Supplementary file 1 [file Table_1.docx]

| Table 1. Comparison of core body temperature before and post intervention in each group ($\bar{X}\pm S$) | | | |
| --- | --- | --- | --- |
| Group | N | Before | Post |
| NC | 10 | 36.96±**0.20** | 36.98±**0.25** |
| AD | 10 | 36.35±**0.78** | 36.33±**0.76** |
| EA | 10 | 36.38±**0.61** | 36.99±**0.31** |
| DD | 10 | 36.39±**0.86** | 36.27±**0.96** |
